# Novel Nitrobenzazolo[3,2-*a*]quinolinium Salts Induce Cell Death through a Mechanism Involving DNA Damage, Cell Cycle Changes, and Mitochondrial Permeabilization

**DOI:** 10.4236/ojapo.2013.22002

**Published:** 2013-04-01

**Authors:** Christian Vélez, Osvaldo Cox, Carlos A. Rosado-Berrios, Dennise Molina, Luz Arroyo, Sujey Carro, Anton Filikov, Vineet Kumar, Sanjay V. Malhotra, Marisol Cordero, Beatriz Zayas

**Affiliations:** 1 Universidad Metropolitana, San Juan, Puerto Rico; 2 Arkansas State University, Jonesboro, USA; 3 University of North Carolina, Chapel Hill, USA; 4 Laboratory of Synthetic Chemistry-SAIC, Frederick National Laboratory for Cancer Research, Frederick, USA; 5 University of Puerto Rico, San Juan, USA

**Keywords:** Apoptosis, A431, Quinolinium Salts, Mutagenesis, Caspase Activation

## Abstract

This study reports the capacity of three nitro substituted benzazolo[3,2-*a*]quinolinium salts NBQs: NBQ 95 (NSC-763304), NBQ 38 (NSC 763305), and NBQ 97 (NSC-763306) as potential antitumor agents. NBQ’s are unnatural alkaloids possessing a positive charge that could facilitate interaction with cell organelles. The anticancer activities of these compounds were evaluated through the National Cancer Institute (NCI) 60 cell line screening which represents diverse histologies. The screening was performed at 10 µM on all cell lines. Results from the NCI screening indicated cytotoxicity activity on six cell lines. In order to explore a possible mechanism of action, a detailed biological activity study of NBQ 95 and NBQ 38 was performed on A431 human epidermoid carcinoma cells to determine an apoptotic pathway involving, cell cycle changes, DNA fragmentation, mutations, mitochondrial membrane permeabilization and caspases activation. DNA fragmentation, cell cycle effects, mutagenesis, mitochondrial permeabilization and activation of caspases were determined by fluorimetry and differential imaging. Our data showed that A431 growth was inhibited with an average IC_50_ of 30 µM. In terms of the mechanism, these compounds interacted with DNA causing fragmentation and cell cycle arrest at sub G_0_/G_1_ stage. Mutagenesis was higher for NBQ 38 and moderate for NBQ 95 Mitochon-drial permeabilization was observed with NBQ 38 and slightly for NBQ 95. Both compounds caused activation of Caspases 3 and 7 suggesting an apoptotic cell death pathway through an intrinsic mechanism. This study reports evidence of the toxicity of these novel compounds with overlapping structural and mechanistic similarities to ellipticine, a known anti-tumor compound.

## 1. Introduction

The development of novel anticancer compounds based on or isolated from natural products has been a productive approach in discovering biologically active compounds [1]. Some of the most promising plant derived products, that have demonstrated activity against cancer cells, are etoposide from genera *Podophyllum* [2,3] ellipticine from the Apocyanaceae family (originally isolated from *Ochrosia elliptica*) [4–6] and berberine from medicinal plants such as e.g. *Berberis aquifolium or Berberis vulgaris* [7]. These substances and their derivatives have been studied for quite some time. Ellipticine and its derivatives are of particular interest to this study because of their structural similarities to the novel compounds reported here, their efficacy against cancer cells, low toxicity in non-cancer cells and less side effects [8].

**Figure 1:**
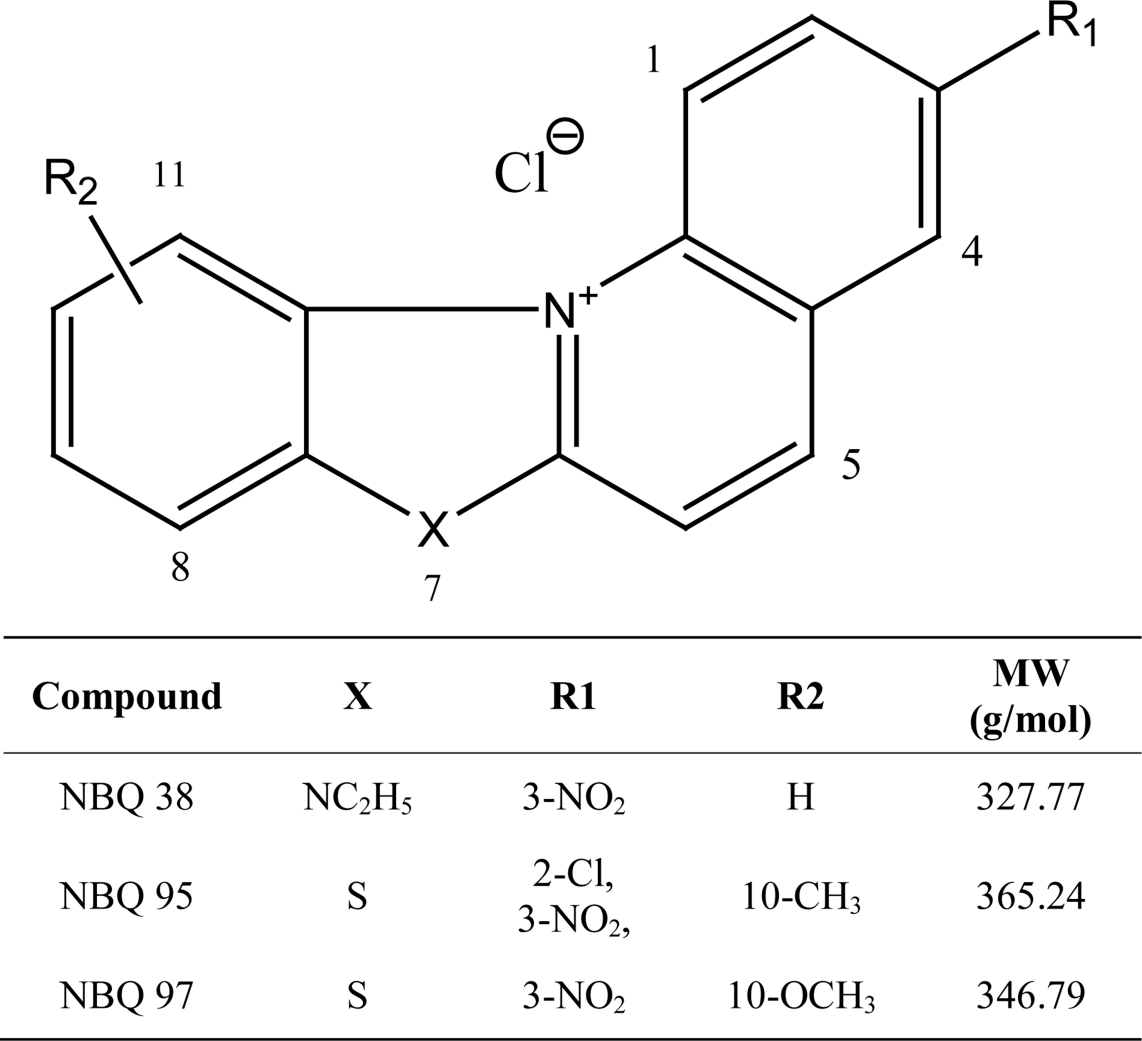
General Structure of three benzazolo[3,2-*a*]quinolinium salts (BQS) reported in this study.

The compounds presented in this study (NBQ 38, NBQ 95, and NBQ 97) belong to a family of unnatural alkaloids known as benzazolo[3,2-*a*]quinolinium salts (BQS). The structural features in this series of compounds shown in Figure 1 include a planar heteroaromatic system incorporating quaternized nitrogen and a fused benzothiazole nucleus. Previously studied members of this family have shown several promising biological properties, for example antitumor activity against P-388 leukemia and Ehrlich ascites in mice [9,10]. Natural compounds with structural similarities overlapping mechanistic similarities to these BQS are berberine and ellipticine. Both compounds have also shown to be apoptosis inducers [6,7,11]. The formation of C8–2’-dG-BQS adducts with calf thymus DNA under hypoxic environment with NBQ-38, one of the compounds presented in this study, has also been reported [12,13].

Similar to ellipticine, NBQ derivatives have shown to form complexes of the intercalative type with DNA [14]. Also, they effectively block DNA as well as RNA and protein synthesis in both KB and Ehrlich ascites cells [10]. The mechanism through which these compounds induce cell damage has not been described until now. Herein, we report the results of the screening of three members of the NBQ family thru the National Cancer Institute’s 60 cell line panel, as well as the evaluation of key apoptotic hallmark events that leads to cell death on A431 epidermoid carcinoma cells as a representative model.

## 2. Materials and Methods

The nitro substituted benzazolo[3,2-*a*]quinolinium salts (NBQ) were synthesized as reported previously [9,12, 15]. Cytogenetically profiled adherent A431 epidermoid-carcinoma cell line was purchased from American Type Culture Collection (ATCC CRL-1555) and grown on RPMI 1640 from Cellgro (Manassas, Va) with glucose and HEPES. Phosphate buffer saline, camptothecin, 6-Thioguanine (6-TG), ethyl methanesulfonate (EMS), hy- poxanthine-aminopterin-thymidine (HAT) and dmethyl sulfoxide (DMSO) were purchased from Sigma Aldrich (St. Louis, MO). DNA fragmentation, mitochondrial per-meabilization and cell cycle reagents were obtained from Chemometec (Allerød, Denmark) and the magic red caspase assay from Immunochemistry Technologies (Bloomington Min).

### 2.1. Instrumentation

Cell quantification was performed using the Countess automated cell counter (Invitrogen, Carlsbad, California). Analysis of DNA fragmentation and cell cycle was performed using the Nucleo counter NC-3000 (Chemometec, Allerød, Denmark) instrument. Caspase activation was measured by fluorimetry analysis using a GloMax^®^-Multi Jr. Single Tube Multimode reader (Promega, Sunnyvale, CA). Characterizations and purity of the experimental NBQ compounds were determined with a Quattro Micro mass spectrometer (Waters Corp, Milford, MA).

### 2.2. BQS and Controls Stock Solutions

For the biological activities assays stock solutions of NBQ 38 and NBQ 95 at 3 mM concentration were prepared with sterile, filtered, deionized water. The stock solutions were kept in sealed glass vials protected from light to avoid photo degradation and stored at 4°C. Biological activity experiments were performed in triplicates. For the NCI 60 cell line screening, compounds (NBQ 38, NBQ 95 and NBQ 97) were sent dry to NCI and samples handled accordingly to NCI’s protocols.

### 2.3. 60 Cell Line Screening

The 3-nitrobenzazolo[3,2-*a*]quinolinium salts *i.e*. NBQ 95 (NSC D-763304), NBQ 38 (NSC: D-763305) and NBQ 97 (NSC: D-763306) were screened through the National Cancer Institute’s Developmental Therapeutics Program [16,17]. The analysis consisted on treating 60 NCI cell lines with each compound at a one dose concentration of 10 µM. Briefly, the panel is organized into nine sub panels representing diverse tissue types: Leukemia, melanoma, and cancers of lung, colon, kidney, ovary, breast, prostate, and central nervous system. The cells are grown in supplemented RPM1 1640 medium for 24 h. The tested compounds were dissolved in DMSO and incubated with cells at 10 µM. The assay is terminated by addition of cold trichloroacetic acid, and the cells are fixed and stained with sulforhodamine B. Bound stain is solubilized, and the absorbance is read on an automated plate reader. The output from the single dose screen is then reported as a mean graph (given in the supporting information) that compares the toxicity of the tested compounds among cell lines.

### 2.4. Cell Viability Inhibition (IC50) Determination

Inhibition of cell viability through the concentration that inhibits 50% of cell growth (IC_50_) was determined for a period of 48 hours. Cultures at a density of 5 × 10^5^ cells were cultured on 12.5 cm^2^ flasks in duplicates and incubated for 4 hours to allow cells to adhere in a normal fashion before being exposed to the tested compounds. Cell cultures contained a total volume of 5 mL including modified RPMI 1640 media (10% FBS) and the tested compound. The negative control was the drug vehicle (sterile water) and the positive control was ellipticine, as a structural analog. Cells were exposed to NBQ 38 and NBQ 95 at concentrations ranging from 0 to 150 µM to determine the IC_50_. After treatment cells were rinsed twice with phosphate buffered saline (PBS), detached with trypLE express (Invitrogen, Carlsbad, California) and counted using the trypan blue exclusion and the Countess automated cell counter. The percent of viable cells was calculated and plotted.

### 2.5. DNA Fragmentation

Fragmentation of DNA as an indication of apoptosis is a commonly used assay in drug-cell interaction studies [18]. This event which is precipitated by nucleases that degrade nucleic acids is quantified using DNA content which measures cells containing less than 1DNA equivalent (Sub-G_1_). The method for the Nucleo counter NC3000 assay is based on the removal of small DNA fragments and retention of DAPI stained higher weight fragments. After treatment with NBQ’s at their respective IC_50_’s and implementing the previously described conditions, cells were harvested, fixed with 70% ethanol, incubated and stained with 1 µg/ml DAPI, according to manufacturer’s specifications and analyzed by image analysis measuring DAPI intensity. For statistical analysis and to assess significance a one way ANOVA with fixed effect was performed. In case significant results were found in the one way ANOVA, a Post Hoc Test Tukey honestly significant difference (HSD) was also performed.

### 2.6. Cell Cycle Effects

Determining the capacity of potential anti-tumor agents to affect or control malignant cell cycle progression is a commonly examined parameter and of great importance on the characterization of their mode of action [19]. Effects on the cell cycle of NBQ 38 and NBQ 95 treated cells was performed with the Nucleo counter NC-3000 instrument which is based on the analysis of DAPI stained cells which indicates the cellular DNA content. After treatment with NBQ’s, cells were harvested, and stained with 10 µg/ml DAPI according to the manufacturer’s specifications and finalizing with analysis of DAPI intensity as an indication of DNA content.

### 2.7. Mutagenesis

The hypoxanthine-guanine phosphoribosyl transferase (HPRT) mutagenesis assay (a somatic cell genetic mutation assay) is used to detect lesions in the DNA after exposure to chemical agents [20–22]. This assay was performed as described by Zayas, *et al*. 2001. In order to reduce the HPRT mutant background level to a minimum, the A431 cells were pre-treated with 1% HAT (hypoxan-thine, aminopterin, and thymidine) selective medium prior to the treatment with NBQ’s compounds. Ethyl me-thanesulfonate (EMS) a known mutagenic and teratogenic compound was used as the positive control and the vehicle (sterilized and filtered water) as the negative control. Surviving cells were considered phenol and genoty-pically normal and were used for NBQ treatment and bulk generation of mutants. A431 cells in monolayer culture were exposed to each NBQ 95 and NBQ 38, at their respective IC_50_s, for a 24 hour period. The treated cultures were maintained in RPMI 1640 growth medium for a period of 5 days to allow near-optimal phenotypic expression of induced mutations. Mutation frequency (MF) was determined by seeding 1 – 2 cells per well in 96 wells plates with medium containing a selective agent, 40 µM of 6-TG to detect mutant cells, and in medium without selective agent to determine the cloning efficiency. After an incubation time of 2 to 3 weeks, the cell colonies were counted. The number of mutant colonies in the selective medium was adjusted by the number of colonies in the nonselective medium, determining the mutation frequency.

### 2.8. Mitochondrial Membrane Permeability

Mitochondrial membrane permeabilization as an indicator of apoptosis induction is a commonly studied parameter in response to disease and stressor substances [23]. Mitochondrial membrane permeabilization was analyzed with applying the mitochondrial potential JC-1 stain assay with the Nucleocounter NC-3000 instrument and following instructions from the manufacturer. Staining solutions used for the assay were; Solution 7 containing 200 µg/ml of JC-1 (5,5’,6,6’-tetrachloro-1,1’,3,3’-tetra-ethylbenzimidazolocarbocyanine iodide) and Solution 8 containing 1 µg/ml DAPI in PBS. Cell cultures of 1 × 10^6^ cells per flask were exposed to each of the NBQs at their respective doses along with the positive control (Valinomycin 10 µM dose) and incubated for 48 hours. After treatment with NBQ’s the cells were rinsed with PBS, detached with 5% trypsin, centrifuged and rinsed with PBS. Samples were stained with the JC-1 reagent then stained with 1 µg/ml DAPI solution, and analyzed immediately. For statistical analysis and to assess significance a one way ANOVA with fixed effect was performed. In case significant results were found in the one way ANOVA, a Post Hoc Test Tukey honestly significant difference (HSD) was also performed.

### 2.9. Caspase Activation

The determination of Caspases 3 and 7 activation which is a hallmark of apoptosis in response to therapeutic drugs [24] was performed by the Magic Red™ assay. This assay monitored the activation of Caspases 3 and 7 using DEVDase, a specific enzyme targeting Caspases 3 and 7. Through this interaction, cells with active Caspases 3 and 7 will present a red fluoresce, while cells with inactive caspases will lack of fluorescence. Stauro-sporine was applied as positive control and drugs’ vehicle as the negative control (water). Cell cultures of 1 × 10^6^ cells were seeded on 25 cm^2^ t-flasks (in duplicates) and treated at their IC_50_ for 48 hours. Following treatments, cells were rinsed with PBS, detached with 5% trypsin, centrifuged and quantified. Aliquots of exposed cells to the nearest 5 × 10^5^ were prepared and stained for an hour following the manufacturer’s instructions with slight modifications. The fluorescence was measured in fluorescence standard units (FSU) by fluorimetry analysis with the GloMax^®^-Multi Jr. single tube multimode reader. Apoptotic cells were estimated by capturing the red fluorescent emission that indicates cleaved MR-[DEVD] at 610 nm. Data was calculated and normalized by subtracting the background emission of the negative control. One way ANOVA with Tukey post test was performed to compare data and determine significance.

## 3. Results

### 3.1. 60 Cell Line Screening

Earlier studies have shown that the quaternary ammonium salts do have inhibitory effect on certain cancer panels [25,26] as seen through NCI 60 cell line screening. Therefore, to investigate the potential anticancer activity of NBQs, these compounds were also passed through single dose testing at the NCI 60 screening panel. The one-dose response data of NBQ 38, NBQ 95 and NBQ 97 is reported as a mean graph of the percent growth of treated cells. The number reported for the one dose assay is growth relative to the no-drug control and relative to the time zero number of cells. This allows detection of both growth inhibition (values between 0 and 100) and lethality (values less than 0). For example, a value of 100 means no growth inhibition effect. A value of 40 would mean 60% growth inhibition effect. A value of 0 means no net growth (or effect) over the course of the experiment. A value of −40 would mean 40% lethality. A value of −100 means all cells are dead. The one-dose data of all the screened compounds is given in Figure 1 (see Supporting Information). A brief summary of the results on selective cell lines where these compounds caused >40% growth inhibition are shown in Table 1. As the data shows, NBQ 95 with electron withdrawing halogen group at Position 2 (next to nitro group) and “S” at position 7 is highly effective in inhibiting the growth of NCI-H522 a Non-small Cell Lung cancer cell line. On replacing “Cl” with “H” (as in NBQ 97) this inhibitory potential is decreased significantly. While, NBQ 38 with no substituent group next to nitrogen and a cyclic amine at Position 7 is highly effective in causing growth inhibition in MDA-MB-468 a Breast cancer cell line. 3-nitrobenzazolo [3,2-*a*]quinolinium salts represent unnatural alkaloids and have some structural similarity with Ellipticine an alkaloid isolated from Apocyanaceae family of plants, which has been reported as an antineoplastic agent, for which the mode of action is considered to be based on DNA intercalation and inhibition of topoisomerase II [27,28]. Motivated with the results of this screen, we embarked upon investigating the mechanism of action of both the NBQ 38 and NBQ 95 compounds, as they demonstrated higher toxicity than NBQ 97.

### 3.2. Cell Viability Inhibition (IC50)

**Figure 2:**
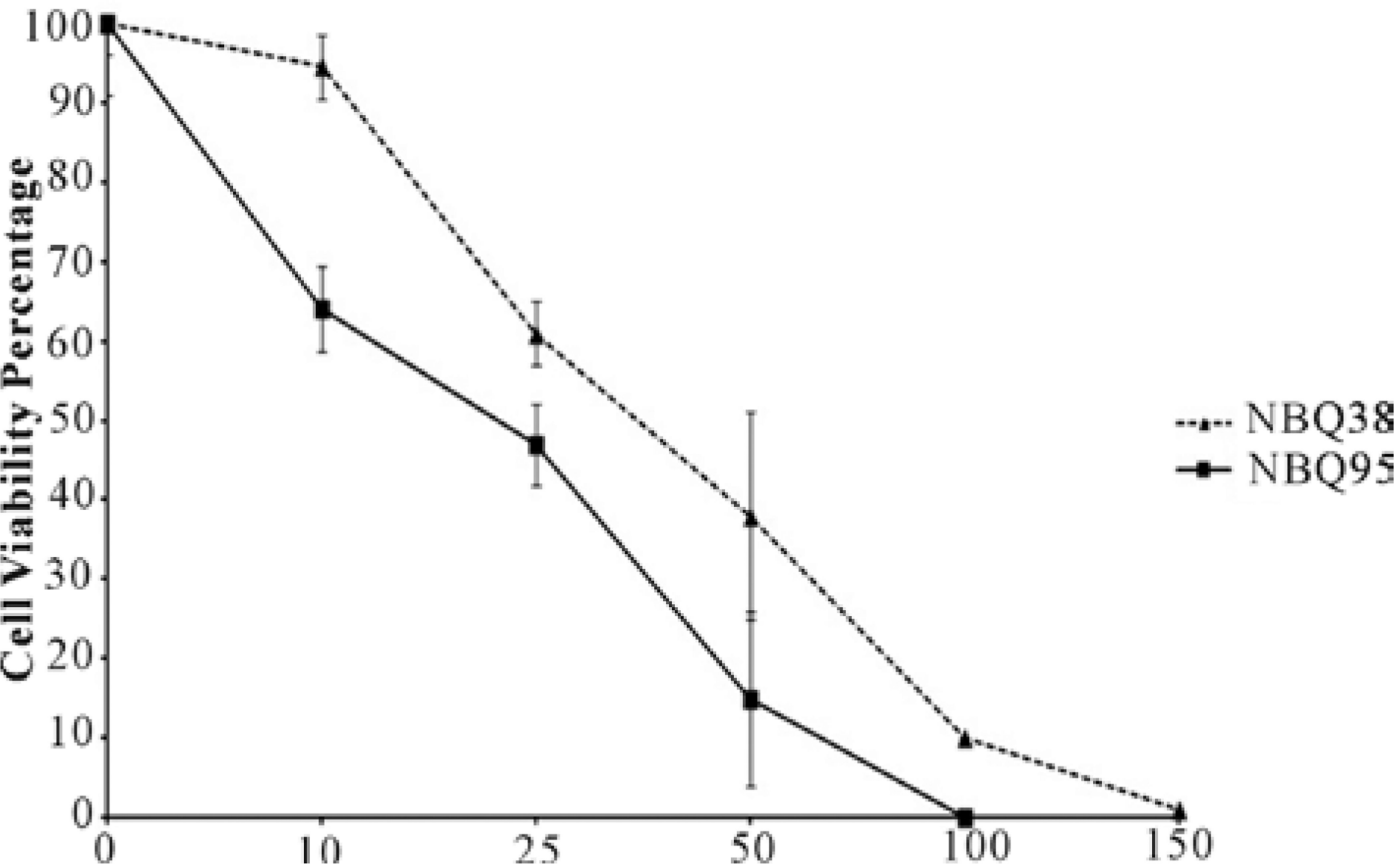
Growth survival analysis of A431 epidermoid carcinoma cells exposed for 48 hours to NBQ 38 and NBQ 95.

The capacity and comparison of the two benzazolo [3,2-*a*]quinolinium salts (BQS) to inhibit cell viability after 48 hours of exposure were evaluated on the A431 epidermoid carcinoma cell line. Cells in culture treated with doses in the range of 10 uM to 150 uM showed a clear dose response with both compounds. The obtained IC_50_ values at 48 hours were 36 µM for NBQ_38_ and 28 µM for NBQ_95_, shown in Figure 2.

### 3.3. DNA Fragmentation

**Figure 3:**
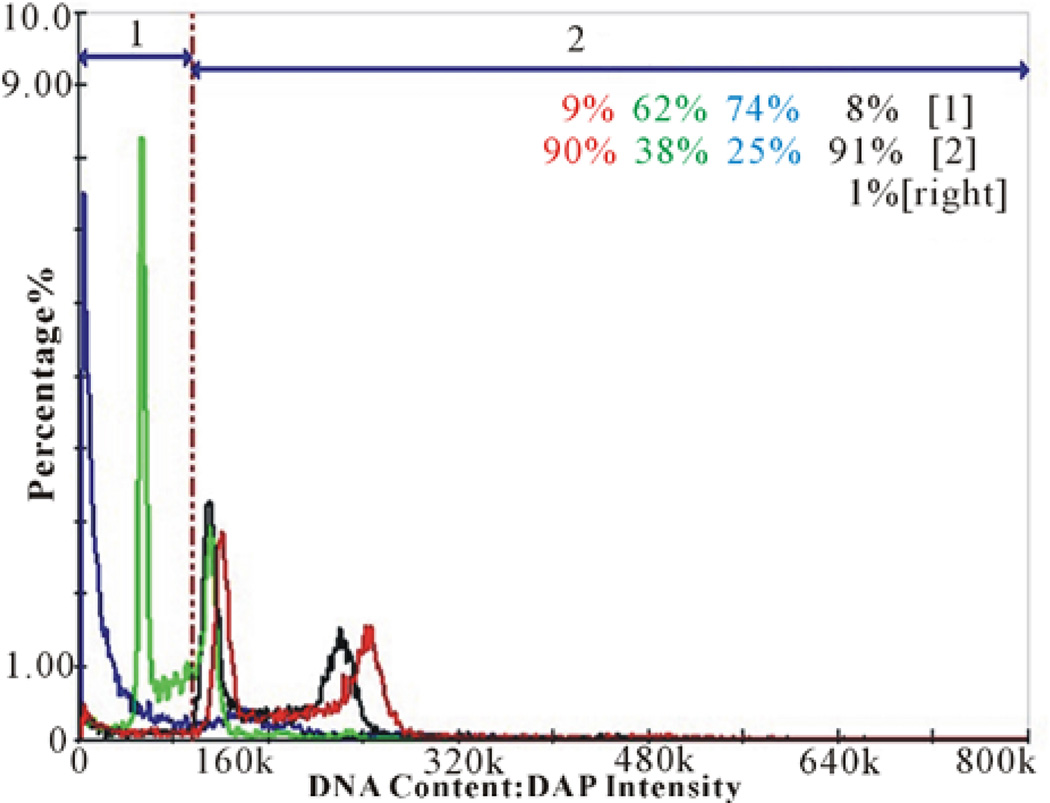
DNA Fragmentation Analysis: A431 cells treated with NBQ 38, NBQ 95 and controls for 48 hours at the IC50 concentration. Peaks display the percentage of fragmented DNA for each sample. Blue peaks (74%) represent the positive control (camptothecin). Black peaks represent the negative control (water) (9%). Green and Red peaks represent the tested compounds, indicating NBQ 38 with a 62% DNA fragmentation and NBQ 95 with 9% DNA fragmentation respectively. Area 1 = fragmented DNA, Area 2 = normal non fragmented DNA. Statistical analysis demonstrated a significant difference (P < 0.05) when NBQ 38 was compared to the DNA fragmentation activity of the negative control, but similar to the positive control. In contrast, NBQ 95 exposed cells presented no significant DNA fragmentation in comparison with the negative control.

DNA fragmentation analysis was performed on A431 cells treated with NBQ 38, NBQ 95, negative control (vehicle) and positive control (camptothecin) for 48 hoursat respective IC50 concentrations determined previously. The display shown in Figure 3 represents the percentage of fragmented DNA for each sample separated in two areas representing the amount of fragmented or non fragmented DNA. Blue peaks (74%) represent the positive control (camptothecin). Black peaks represent the negative control (water) (9%). Green and Red peaks represent the tested compounds, NBQ 38 (62%) and NBQ 95 (9%) respectively. Area 1 = fragmented DNA, Area 2 = normal non-fragmented DNA. Results clearly indicate that NBQ 38 was the most active compound with the highest percentage of fragmented DNA (62%). NBQ 95 was less active with only 9% of cells with fragmented DNA as indicated in Region 1 (Figure 3). NBQ 38 presented a significant difference (P < 0.05) when com-50 pared to the negative control but similar to the positive control. In contrast, NBQ 95 exposed cells presented no significant DNA fragmentation in comparison with the negative control. The results clearly demonstrate that NBQ 38 exposed cells undergo significant DNA fragmentation after 48 hour exposure similar to camptothecin derivatives [29, 30], a known anti-tumor drug.

### 3.4. Cell Cycle Effects

**Figure 4:**
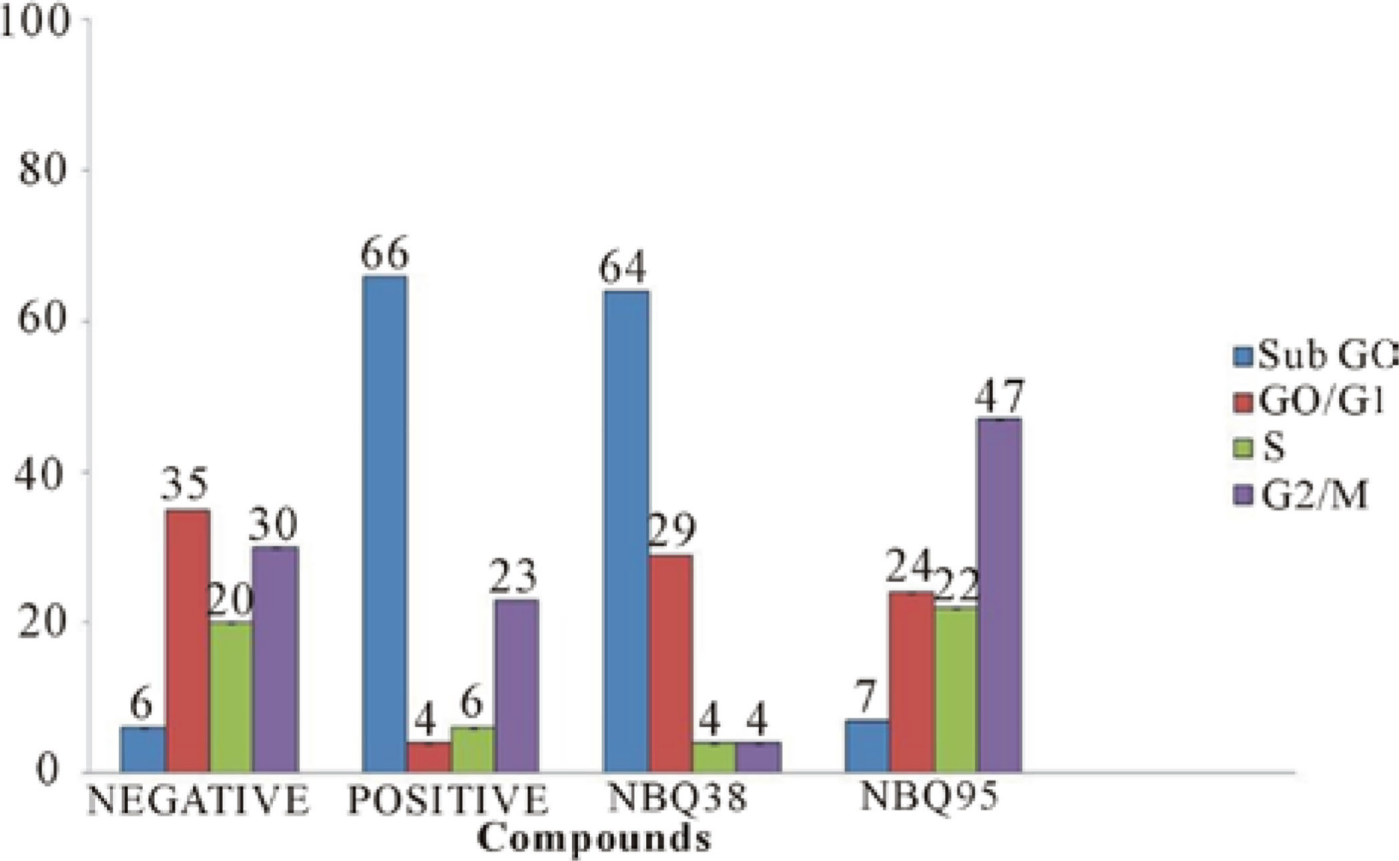
Cell cycle effects on A431 cells treated with NBQ 38 NBQ 95, camptothecin (positive control) and vehicle (water) as negative control after 48 hours’ exposure. Blue column indicates sub G0 cells, red G0/G1, green S phase and purple G2/M.

After a 24 hour exposure to the NBQs cells were analyzed for cell cycle effects. Results indicated that NBQ 38 was the most active compound causing (64%) cell cycle arrest at sub G_0_/G_1_. Most of these cells did not reach the G_2_/M phase and only 29% reached the G_0_/G_1_ stage. Meanwhile, NBQ 95 treated cells showed low arrest at G_0_/G_1_ but a high percentage (47%) at G_2_/M phase (Figure 4). The prevalence of cells at G_0_/G_1_ suggest that overall both compounds cause arrest of the cell cycle even though there is a difference at the cell cycle stage where the majority of cells are found per compound. NBQ 38 presents clearly similarities with the positive control camptothecin in their capacity to arrest cells at sub G_0_/G_1_ [31, 32].

### 3.5. HPRT Assay

**Figure 5:**
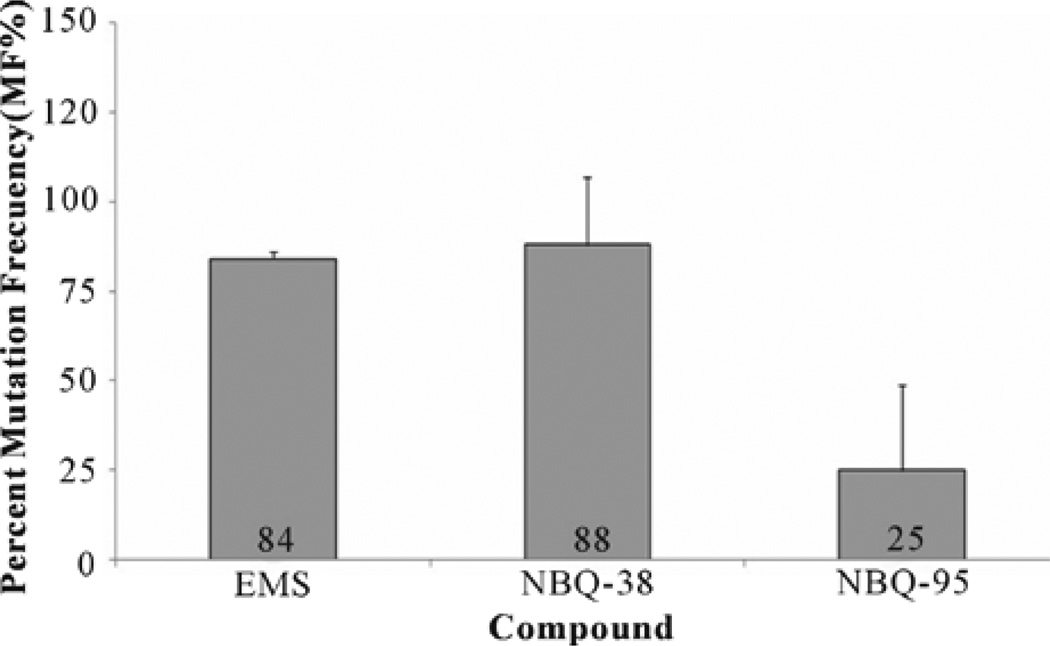
HPRT Gene Mutational Assay: HAT pre-treated A431 cells were exposed to NBQ 38, NBQ 95 and to the positive control Ethyl methanesulfonate (EMS) for 24 hours, allowed to recover for 5 days then continuously exposed to 40 µM 6-TG to select for induced mutants. After 2 – 3 weeks formed colonies were counted. The results were measured in mutation frequencies (MF). Observed degree of mutagenicity was as follows: NBQ 38 (MF 88%), EMS (MF 84%), and NBQ 95 (MF 25%).

The HPRT assay permits assessment of the NBQ’s interaction with DNA and their capacity to induce mutation altering the genetic information. As expected the positive control EMS (50 µg/ml) presented a high Mutation Frequency (MF) of 84% + 19. Similar to the EMS the experimental NBQ 38 presented a high MF of 88% + 24 however, NBQ 95 induced a much lower MF of 25% + 12 (Figure 5). Thus, NBQ 38 showed to be the most active mutagenic compound with over 63% higher mutagenesis induction in contrast to NBQ 95, suggesting it to be highly reactive with DNA as also seen with the known mutagenic positive control EMS [33].

### 3.6. Mitochondrial Membrane Permeability

**Figure 6:**
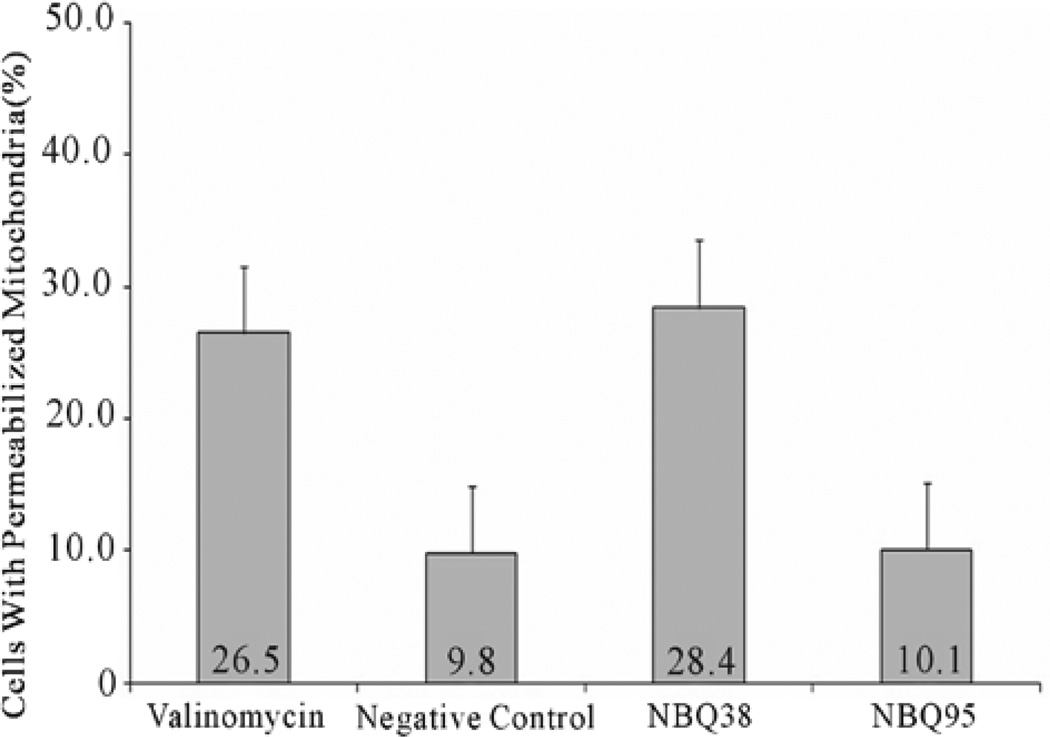
Mitochondrial Membrane Permeabilization: Data is shown in percentage of permeabilized cells. Results present the permeabilization capacity of NBQ 38 (28.4%) to be slightly higher than the positive control, valinomycin (26.5%). NBQ 95 re- ported the lower permeabilization percentage (10.1%) comparable to the negative control (9.8%). Statistical analysis proved significant difference (P < 0.05) between the negative control and NBQ 38.

**Figure 7:**
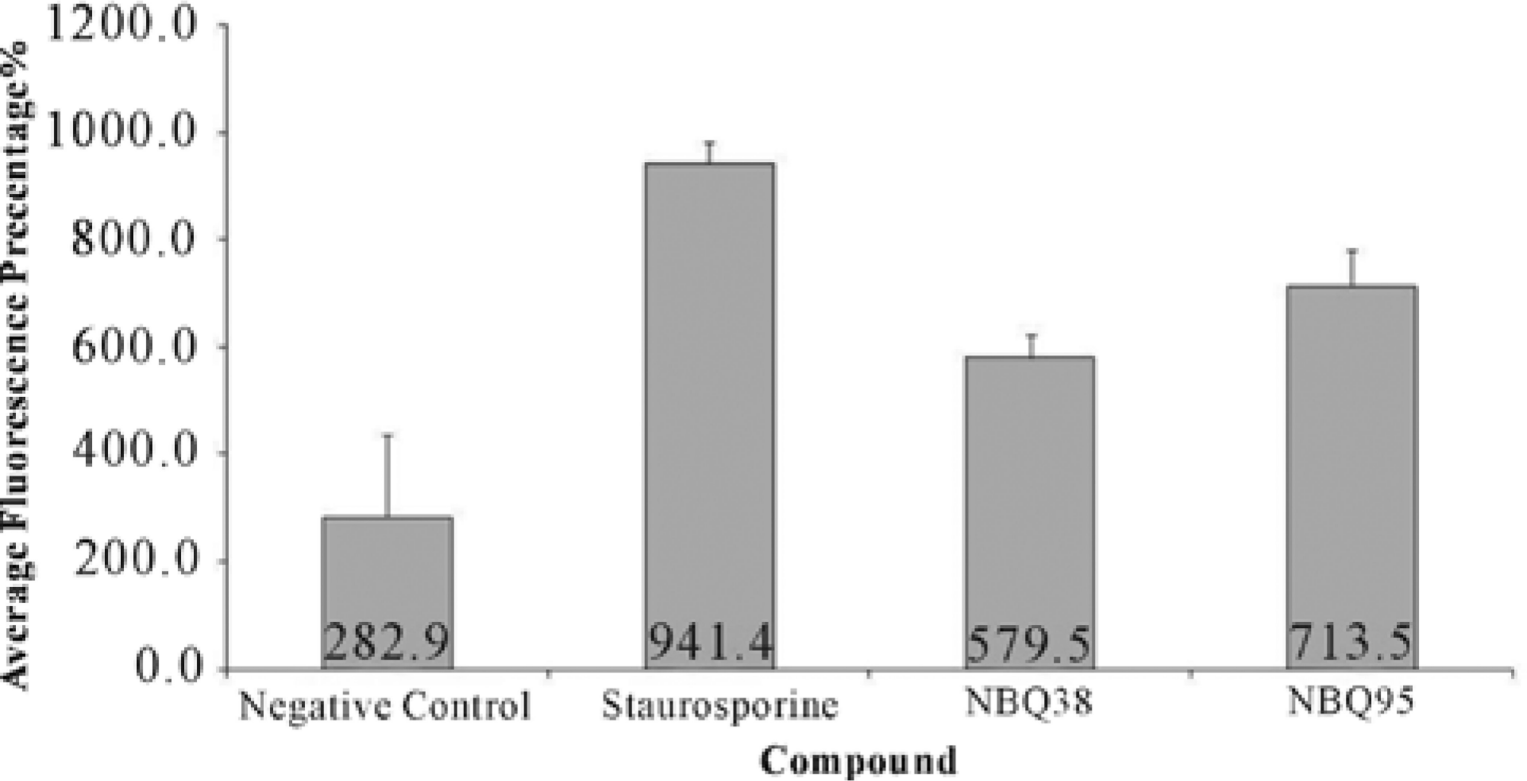
Caspase 3 and 7 activation: Data is shown in average standard fluorescence units (FSU). A431 cells treated with NBQ 38, NBQ 95, staurosporine (positive control) and vehicle. Results indicate staurosporine (positive control) induces the highest Caspases 3 and 7 activation with an average FSU of 941.44 ± 69.17 followed by NBQ 95 (713.45 ± 41.87 FSU), NBQ 38 (579.52 ± 39 FSU) and negative control (282.89 ± 153.5 FSU). Statistical analysis proved no significant difference (P < 0.05) between the positive control and the tested compounds. Significant difference was observed in contrast to the negative control.

Mitochondrial membrane permeabilization (MMP) is a well-known hallmark of cellular stress and apoptosis [34,35]. MMP as an indicator of apoptosis was analyzed with the Nucleo counter NC-3000 instrument following manufacturer’s mitochondrial potential JC-1 assay. Results demonstrated higher permeabilization (28.4%) on NBQ 38 exposed cells (Figure 6), which is comparable to26.5% for the valinomycin positive control (P < 0.05). NBQ 95 presented lower value (10.1%), which is comparable to 9.8% for the negative control P compounds NBQ 95 (713.45 ± 41.87 FSU) presented the highest caspases activation followed by the NBQ 38 (579.52 ± 39 FSU) (Figure 7). Both experimental compounds presented com- parable values to the positive control (P < 0.05). The over- all quantitative analysis suggests activation of Caspases 3 and 7 and indicates their participation in an apoptosis induction process of A431 tumor cells exposed to the NBQs.

## 4. Discussion

This study describes the cell viability inhibitory activity of three novel unnatural alkaloids nitro substituted benzazolo[3,2-*a*]quinolinium salts toward the NCI 60 cell panel as well as a general evaluation of their potential mode of action. Herein, we also report the biological capacity of the two most active compounds to induce DNA fragmentation and mutation, produce cell cycle arrest and cause cell death in an apoptotic fashion involving mitochondrial membrane depolarization and activation of Caspases 3 and 7.

The results from the NCI 60 cell line screening panel based on a one dose (10 µM) analysis are shown in Table 1. Both compounds show 40% to 93% growth in hibition capacity on 6 specific cell lines (10%) of the tested cell lines. These cell lines represent various tissues of origin including: lung, colon, central nervous system, ovarian and breast. NBQ 95 showed strong growth inhibition (93%) on the non-small cell carcinoma cells (NCI-H522) followed by a 59% growth inhibition on the MDA-MB-468 cancer cells. In contrast NBQ 38 affects higher cell survival on MDA-MB-468 breast cancer cell line (89%), followed by a 60% growth inhibition on the OVCAR-8 ovarian cancer cell line. The third compound tested, NBQ 97 reported less overall activity in all cell lines.

The broad spectrum of tissue types that reported susceptibility to both NBQ 95 and NBQ 38 guided us into evaluating biological activities that could provide insights into the mode of action of these compounds. The 60 cell lines on the NCI screening panel have varied expression level of the epidermal growth factor receptor (EGFR) and p53 genes. While, A431 cells express abnormally high levels of the EGFR and contain no functional p53. As such, these two genes are the differentiating factors and their expression levels in A431 cells appear to represent similarity to many of the cell lines in the NCI panel in which our compound show activity. Therefore, the mechanistic study was carried with A431 cells. The structural similarities of NBQ 38 and NBQ 95 with ellipticine motivated us to consider and analyze the capacity of these agents to interact with DNA, cause cell cycle arrest and apoptosis induction including changes to mitochondrial membrane potential and caspases activation.

The cell viability inhibition analysis on A431 cells at doses ranging from 10 µM to 150 µM, reported IC_50_ concentrations of 36 µM and 28 µM for NBQ 38 and NBQ 95 respectively and although lower than those reported for ellipticine, in other cell lines (up to 10 µM) [36,37] we consider this to be in close (µM) range. In terms of the mode of action similarities where also observed between ellipticine and the NBQs. Ellipticine’s cytotoxic involves mutagenic effects and DNA damage including intercalation, adduct formation and inhibition of topoisomerase II [38,39]. The representative NBQ’s studied here show the ability to induce cell cycle arrest and DNA fragmentation, with stronger effects being observed in cells exposed to NBQ 38, which caused arrest at G_0_/G_1_ similarly to what has been reported for ellipticine [40,41]. This cell arrest may lead eventually to activation of apoptotic mechanisms that cause mitochondrial membrane permeabilization and caspase activation [42,43]. Another piece of evidence for BQS-DNA interaction is the fragmentation of DNA, which was clearly evident and significant with NBQ 38, and at a lesser degree with NBQ 95. Mutation induction was also observed with both compounds. The observed mutation frequencies were high with NBQ 38 and moderate for NBQ 95. The combinations of the evaluated biological activities suggest a nuclear promoted mechanism of cell death. The ability of these compounds especially NBQ 38 to promote DNA alterations also confirms results from previous studies in the formation of 2’-dG-NHBQ 38 adducts in naked DNA were detected [12].

DNA damage is known to provoke a downstream effect in which the depolarization of mitochondrial membrane and activation of effector caspases, are the most probable events suggesting an apoptotic pathway for cell death [44]. This has been confirmed in the current study as seen with NBQ 38 exposed cells which cause mitochondrial membrane permeabilization, while both NBQ 38 and NBQ 95 exposed cells cause activation of Caspases 3 and 7. It is important to mention that the A431 cells used in this study are p53 deficient which suggest that the intermediate steps after DNA damage could be mediated through another pathway such as p73 [45]. This p73 activation pathway has been observed for the NBQ’s structural analog ellipticine [46] where ellipticine and a derivative caused DNA damage through intercalation involving activation of page 73 pathway even through a DNA damage independent matter. This evidence suggests that ellipticine or its analogues can activate p53 and/or p73 pathways.

As reported in the literature [47,48] another possible cellular event triggering apoptosis and considering the observed mitochondrial membrane alteration could involve stress induced by NBQ’s to the endoplasmic reticulum. When the endoplasmic reticulum is stressed, Bcl-2 mediated mitochondria permeabilization [49], leading to the opening of the mitochondrial permeability transition pore (PTP) [50] and activation of caspase cascade ultimately ending in cell death. This pathway although possible has not been tested for these Novel NBQs.

## 5. Conclusion

In conclusion, this study reports the effects of NBQ 38, NBQ 95, and NBQ 97, three novel ellipticine analogues, on the NCI 60 cell line panel with toxicities of 40% to 93% in cells from varies tissue types. Furthermore, the possible mechanism of action of NBQ 38 and NBQ 95 has been studied with A431 cells. These two drugs demonstrated cytotoxicity comparable to its structural analogue, ellipticine including overlapping mechanisms of action. The results show that these compounds interacted with DNA causing effects such as mutagenesis as well as cell cycle arrest followed by mitochondrial membrane depolarization and the activation of Caspases 3 and 7 and DNA fragmentation.

## Supplementary Material

N38

N95

## Figures and Tables

**Table 1:** Summary of selected cell lines from the NCI 60 cell lines creening panel treated with NBQ 38, NBQ 95 and NBQ 97 resulting in over 40% viability inhibition at a 10 micro mlar concentration.

Panel	Cell Iine	% Inhibition		
		Nbq 95 D-763304	NBQ 38 D-763305	NBQ 97 D-763306
Non-small cell lung cancer	NCI-H5 22	93	>40	>40
Colon cancer	KM12	>40	>40	53
Central nervous system cancer	SF-268	>40	51	46
Ovarian	OVCAR-3	>40	40	>40
Cancer	OVCAR-8	>40	60	>40
*Epidermoid carcionoma	A431	40	5.5	N/A

* Non panel assay.
