# Supplementary material for: Novel Nitrobenzazolo[3,2-a]quinolinium Salts Induce Cell Death through a Mechanism Involving DNA Damage, Cell Cycle Changes, and Mitochondrial Permeabilization
Source: Open J Apoptosis. Author manuscript; Available in PMC 2014 Sep 19. (PMC4169051; doi:10.4236/ojapo.2013.22002)

Developmental Therapeutics Program
One Dose Mean Graph

NSC: D-763305 / 1	Conc: 1.00E-5 Molar	Test Date: Jan 09, 2012
Experiment ID: 1201OS93		Report Date: Feb 08, 2012

Panel/Cell Line	Growth Percent
**Leukemia**	
HL-60(TB)	85.67
K-562	74.24
MOLT-4	75.63
RPMI-8226	71.99
SR	86.73
**Non-Small Cell Lung Cancer**	
A549/ATCC	91.58
EKVX	67.16
HOP-62	91.08
HOP-92	90.03
NCI-H226	105.76
NCI-H23	72.27
NCI-H460	90.20
NCI-H522	77.02
**Colon Cancer**	
COLO 205	88.18
HCC-2998	106.14
HCT-116	90.55
HCT-15	101.08
HT29	75.83
KM12	75.55
SW-620	87.48
**CNS Cancer**	
SF-268	48.68
SF-295	106.54
SF-539	76.36
SNB-19	91.55
SNB-75	99.21
U251	83.51
**Melanoma**	
LOX IMVI	92.13
MALME-3M	61.26
M14	114.55
MDA-MB-435	108.19
SK-MEL-2	105.98
SK-MEL-28	106.21
SK-MEL-5	83.70
UACC-257	90.71
UACC-62	107.12
**Ovarian Cancer**	
IGROV1	97.13
OVCAR-3	59.96
OVCAR-4	70.75
OVCAR-5	106.07
OVCAR-8	39.85
NCI/ADR-RES	104.32
SK-OV-3	103.89
**Renal Cancer**	
786-0	111.34
A498	71.66
ACHN	95.69
CAKI-1	100.22
RXF 393	109.92
SN12C	95.44
UO-31	89.28
**Prostate Cancer**	
PC-3	91.11
DU-145	95.93
**Breast Cancer**	
MCF7	100.50
MDA-MB-231/ATCC	78.91
HS 578T	116.48
BT-549	112.62
T-47D	85.09
MDA-MB-468	10.65
Mean	88.19
Delta	77.54
Range	105.83

Mean Growth Percent - Growth Percent

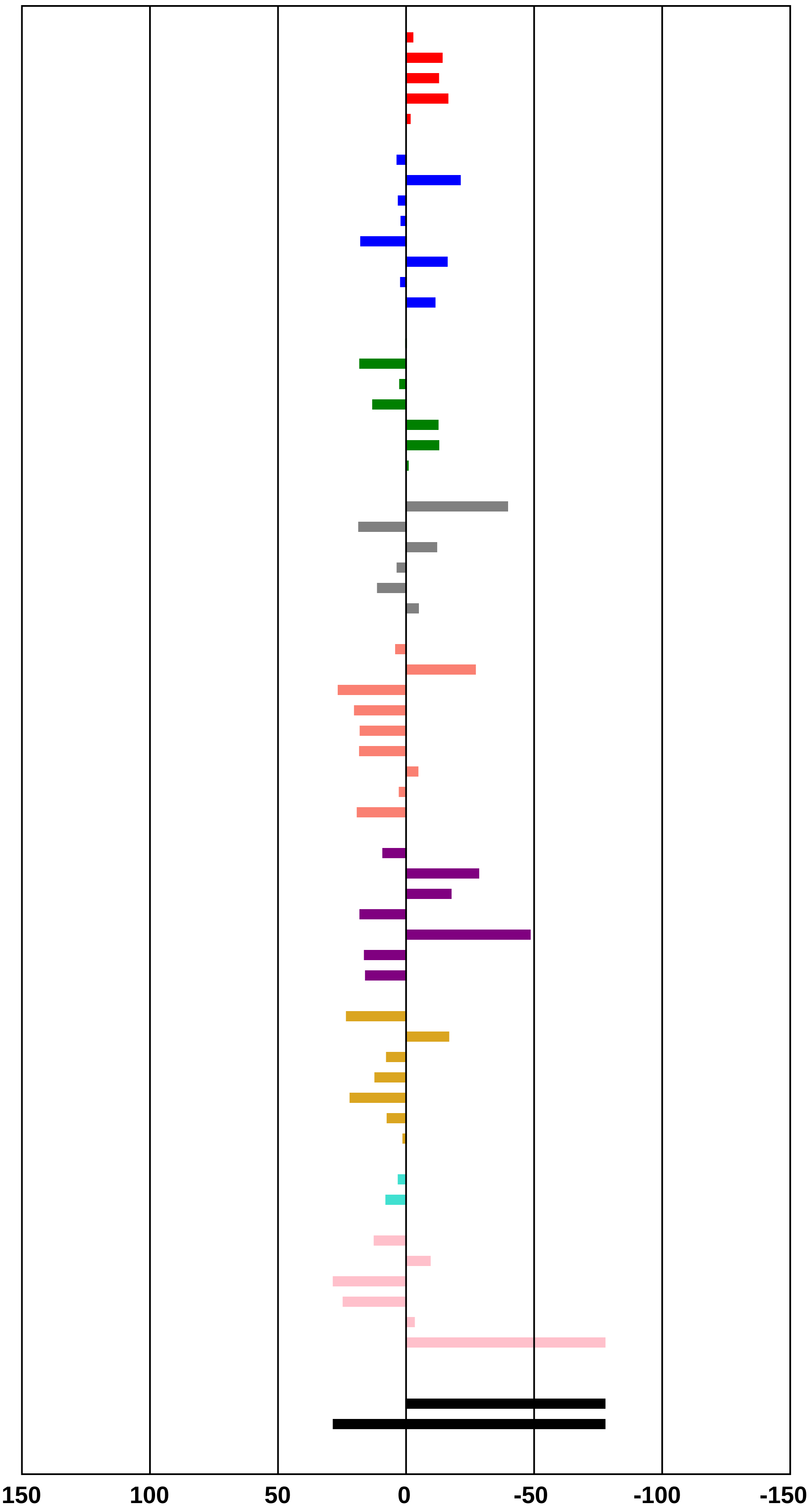

Supplement: N38 [file NIHMS579234-supplement-N38.pdf]
