# Supplementary material for: Novel Nitrobenzazolo[3,2-a]quinolinium Salts Induce Cell Death through a Mechanism Involving DNA Damage, Cell Cycle Changes, and Mitochondrial Permeabilization
Source: Open J Apoptosis. Author manuscript; Available in PMC 2014 Sep 19. (PMC4169051; doi:10.4236/ojapo.2013.22002)

Developmental Therapeutics Program
One Dose Mean Graph

NSC: D-763304 / 1 | **Conc:** 1.00E-5 Molar | **Test Date:** Jan 09, 2012

Experiment ID: 1201OS93 | **Report Date:** Feb 08, 2012

Panel/Cell Line	Growth Percent
**Leukemia**	
CCRF-CEM	101.01
HL-60(TB)	103.76
K-562	83.82
MOLT-4	100.09
RPMI-8226	95.00
SR	92.19
**Non-Small Cell Lung Cancer**	
A549/ATCC	104.12
EKVX	105.22
HOP-62	132.80
NCI-H226	122.03
NCI-H23	103.34
NCI-H460	90.25
NCI-H522	6.57
**Colon Cancer**	
COLO 205	107.14
HCC-2998	94.80
HCT-116	101.35
HCT-15	100.65
HT29	99.70
KM12	92.12
SW-620	100.80
**CNS Cancer**	
SF-268	105.31
SF-295	111.61
SF-539	103.35
SNB-19	93.50
SNB-75	92.84
U251	87.97
**Melanoma**	
LOX IMVI	95.38
MALME-3M	112.50
M14	118.73
MDA-MB-435	100.58
SK-MEL-2	91.84
SK-MEL-28	109.03
SK-MEL-5	84.92
UACC-257	106.16
UACC-62	91.69
**Ovarian Cancer**	
IGROV1	82.82
OVCAR-3	89.67
OVCAR-4	86.25
OVCAR-5	111.92
OVCAR-8	99.88
NCI/ADR-RES	104.55
SK-OV-3	81.92
**Renal Cancer**	
786-0	102.75
A498	74.84
ACHN	105.40
CAKI-1	109.75
RXF 393	121.28
SN12C	96.48
UO-31	109.71
**Prostate Cancer**	
PC-3	90.82
DU-145	98.36
**Breast Cancer**	
MCF7	92.13
MDA-MB-231/ATCC	77.04
HS 578T	99.46
BT-549	138.87
T-47D	94.81
MDA-MB-468	40.64
Mean	97.39
Delta	90.82
Range	132.30

Mean Growth Percent - Growth Percent

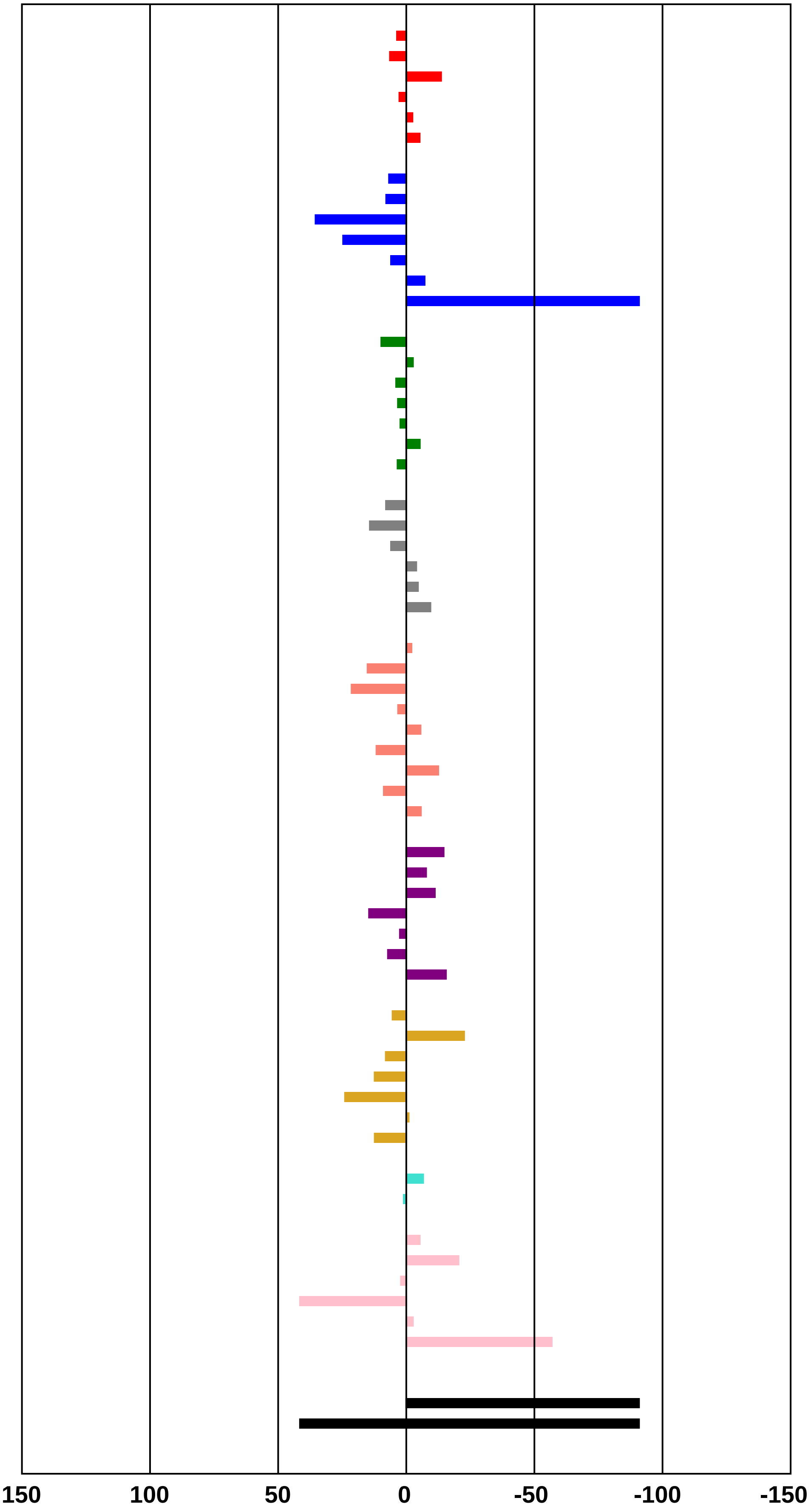

Supplement: N95 [file NIHMS579234-supplement-N95.pdf]
